# Localization of lipopolysaccharide from Escherichia Coli into human atherosclerotic plaque

**DOI:** 10.1038/s41598-018-22076-4

**Published:** 2018-02-26

**Authors:** Roberto Carnevale, Cristina Nocella, Vincenzo Petrozza, Vittoria Cammisotto, Luca Pacini, Veronica Sorrentino, Ombretta Martinelli, Luigi Irace, Sebastiano Sciarretta, Giacomo Frati, Daniele Pastori, Francesco Violi

**Affiliations:** 1 Department of Medical-Surgical Sciences and Biotechnologies, Sapienza University of Rome, Latina, Italy; 2 I Medical Clinic, Atherothrombosis Center, Department of Internal Medicine and Medical Specialties, Sapienza University of Rome, Rome, Italy; 3 Unit of Vascular Surgery, Department “Paride Stefanini”, Sapienza University of Rome, Rome, Italy

## Abstract

Experimental studies showed that gut-derived lipopolysaccharide (LPS) is pro-atherogenic, however, its relationship with human atherosclerosis is still to be defined. We investigate if gut-derived LPS from *Escherichia Coli* localizes in human carotid plaque and its potential role as pro-inflammatory molecule in the atherosclerotic lesion. LPS from *Escherichia Coli* and Toll-like receptor 4 (TLR4) were studied in specimens from carotid and thyroid arteries of 10 patients undergoing endarterectomy and 15 controls matched for demographic and clinical characteristics. Blood LPS were significantly higher in patients compared to controls. Immunochemistry analysis revealed positivity for antibodies against LPS and TLR4 coincidentally with positivity for CD68 only in the atherosclerotic plaque of carotid arteries but not in thyroid arteries; the positivity for LPS and TLR4 was greater in the area with activated macrophages. LPS concentration similar to that detected in atherosclerotic plaque resulted in a dose-dependent TLR4-mediated Nox2 up-regulation by human monocytes. These data provide the first evidence that LPS from *Escherichia Coli* localizes in human plaque and may contribute to atherosclerotic damage via TLR4-mediated oxidative stress.

## Introduction

Recent experimental and clinical studies discovered that intestinal microbiota is implicated in the atherosclerotic process^1^. For example, trimethylamine N-oxide, which stems from microbial conversion of dietary nutrients containing choline, phosphatidylcholine and L-carnitine, possesses pro-atherogenic and pro-thrombotic activities and is associated with cardiovascular risk^2^. Other products of gut microbiota, such as LPS, which is a major component of the outer layer of Gram-negative bacteria, may also be involved in the athero-thrombosis process. In the circulation of healthy subjects LPS may be found in a range of concentration of approximately 15–200 pg/ml^3^. One mechanism through which LPS enters human circulation is with apolipoprotein B48-containing lipoproteins, therefore increasing after a lipid-rich meal^4^. In addition, LPS may cross gastro-intestinal tube because of increased gut permeability due to impaired tight junctions. This phenomenon occurs in several conditions associated with atherosclerosis, such as type II diabetes, obesity and hypertension, which may display elevated LPS circulating levels^5^. The pro-atherogenic role of LPS is supported by experiments showing smaller atherosclerotic plaque in animals with genetic deletion of TLR4^6^ and acceleration of plaque formation in animals injected with LPS^7^. A hypothetical relationship between LPS and human atherosclerosis has been suggested by a prospective study that documented an association between circulating LPS and atherosclerosis burden^8^. Moreover, LPS may contribute to the thrombotic process, even at concentrations found in the human circulation; thus, a recent study demonstrated that LPS amplifies the platelet response to common agonists upon interaction with its receptor TLR4^9^. However, the impact of gut-derived LPS in human atherosclerotic plaque is still to be clarified. We speculated that, once reached systemic circulation, LPS might cross endothelial wall eliciting a pro-inflammatory response upon cell interaction within the sub-endothelial space.

## Results

Demographic and clinical variables in patients and controls are reported in the Table 1. There were no differences in terms of age, gender and concomitant anti-atherosclerotic and anti-thrombotic treatment. Compared to patients who were complicated by >70% critical stenosis, controls had no signs of carotid atherosclerotic plaque by Doppler ultrasonography. Furthermore, neither patients nor controls displayed signs of acute or chronic infection.

**Table 1:** Clinical and laboratory characteristics of patients and controls.

	Controls (n = 15)	Patients (n = 10)	p
Age	70.7 ± 6.0	73.1 ± 8.9	0.433
Women (%)	26.7	20.0	0.702
Hypertension (%)	100.0	100.0	—
Dyslipidaemia (%)	90	90	—
Diabetes (%)	53.3	30.0	0.414
BMI (Kg/m^2^)	28.2 ± 2.7	27.1 ± 2.6	0.458
ACE/ARBs (%)	86.7	70.0	0.358
Β-blockers (%)	33.3	10.0	0.345
Antiplatelet drugs (%)	100.0	100.0	—
Statins (%)	100.0	90.0	0.400
Serum LPS (pg/ml)	43.5 ± 11.9	79.0 ± 10.7	<0.001
Serum zonulin (ng/ml)	2.2 ± 1.1	4.5 ± 0.7	<0.001

Circulating levels of LPS (79.0 ± 10.7 vs. 43.5 ± 11.9 pg/ml, p < 0.001) and zonulin (4.5 ± 0.7 vs. 2.2 ± 1.1 ng/ml, p < 0.001) were significantly higher in patients compared to controls (Table 1). Circulating LPS correlated with serum zonulin (r = 0.553, p < 0.05) and soluble TLR4 (r = 0.490, p < 0.05).

**Figure 1:**
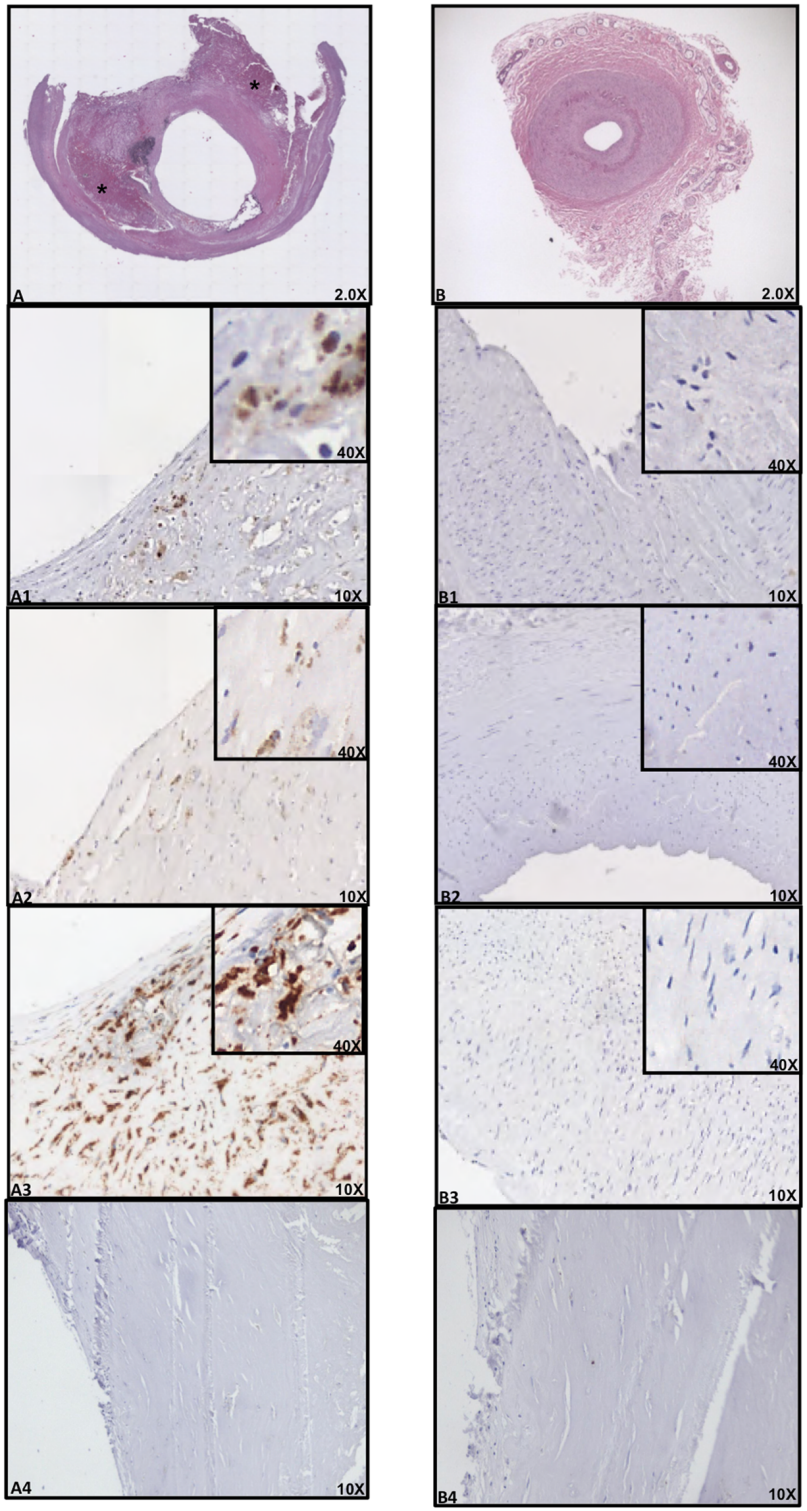
Immune-histochemical (IHC) analyses of LPS, TLR4 and CD68 in carotid plaques and superior thyroid arteries. Immunostaining for LPS and TLR4 and cell phenotype in carotid atherosclerotic plaque (**A**) and thyroid artery (**B**). Serial sections (2 μm) were prepared as described in Online Methods and stained with hematoxylin and eosin. The presence of the antigen recognized by the primary antibody is indicated by a brown substrate. IHC analysis revealed immunoreactivity for antibodies against LPS from *Escherichia Coli* (EC) (A1, 10X), TLR4 (**A2**, 10X) and CD68 (**A3**, 10X) in three serial sections of carotid plaque particularly in the area associated with necrotic lipid core (asterisk). Immunostaining for anti-LPS, anti-TLR4 and anti-CD68 reveals no reactivity in thyroid arteries (**B1–B3**, 10X). Negative control, with a rabbit preimmune serum, in the area of the plaque near the lipid necrotic core and thyroid artery section **(A4** and **B4**, 10X**)**. A view of Panels A1–A3 and Panels B1–B3 at higher magnification was reported (40X).

**Figure 2:**
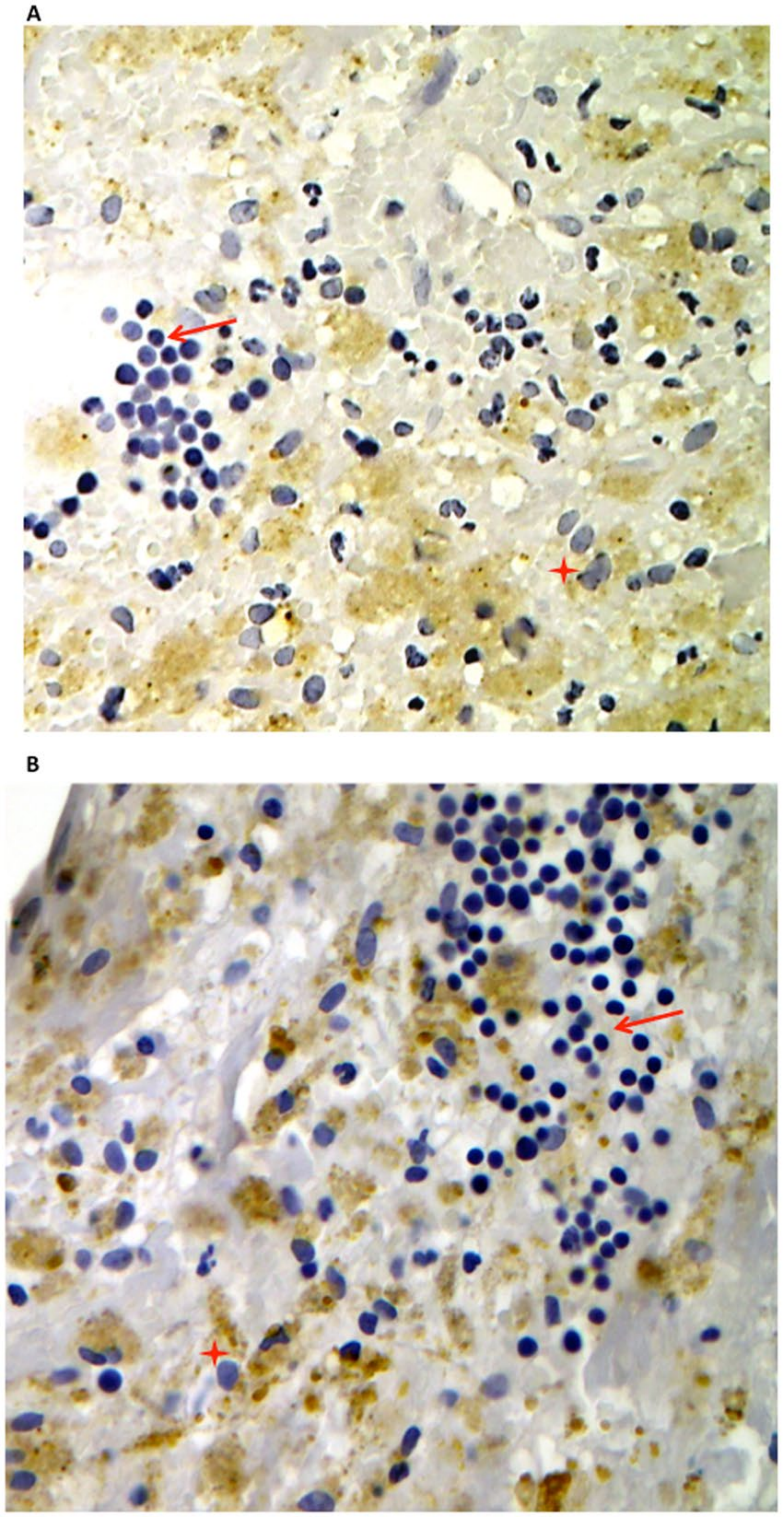
Morphological changes of activated macrophages. Immunostaining for LPS (**A**) and TLR4 (**B**) in carotid plaque revealed that in the area where the immunoreactivity for antibodies against LPS from *Escherichia Coli* (EC) (**A**) or for antibodies against TLR4 (**B**) was higher, macrophages CD68 positive showed signs of activation (red star). They appeared bigger compared to non-activated macrophages (arrow), which, conversely, were detected in the area with a less reactivity to LPS and TLR4 and disclosed typical round shape.

Immuno-histochemical analysis revealed the presence of an atheromatous core with foam cells and lymphocytes close to the lipid core in all carotid arteries (Fig. 1A). Conversely, thyroid arteries did not disclose any sign of atherosclerotic damage (Fig. 1B). Moreover, hematoxylin and eosin staining of the carotid plaque revealed the presence of calcium precipitates at the level of the media tunica of carotid artery, which were associated with cholesterol crystals and inflammatory lympho-mononuclear cells. In three serial sections of this area, IHC analysis revealed immunoreactivity for antibodies against LPS from *Escherichia Coli* (EC) (Fig. 1A1), TLR4 (Fig. 1A2) and CD68 antibody (Fig. 1A3) in all the carotid samples, while no IHC reactivity was detected in thyroid arteries (Fig. 1B1–B3). Serial sections stained with CD68 antibody were also positive for antibodies against LPS (37%) and TLR4 (33%) (Fig. 1A1–A3). In the carotid plaque sections, analysis of macrophage morphology showed that, in the area positive for LPS and TLR4, macrophages were much bigger (Fig. 2A,B, star) compared to macrophages in the area with less reactivity to LPS and TLR4 (Fig. 2A,B, arrow).

**Figure 3:**
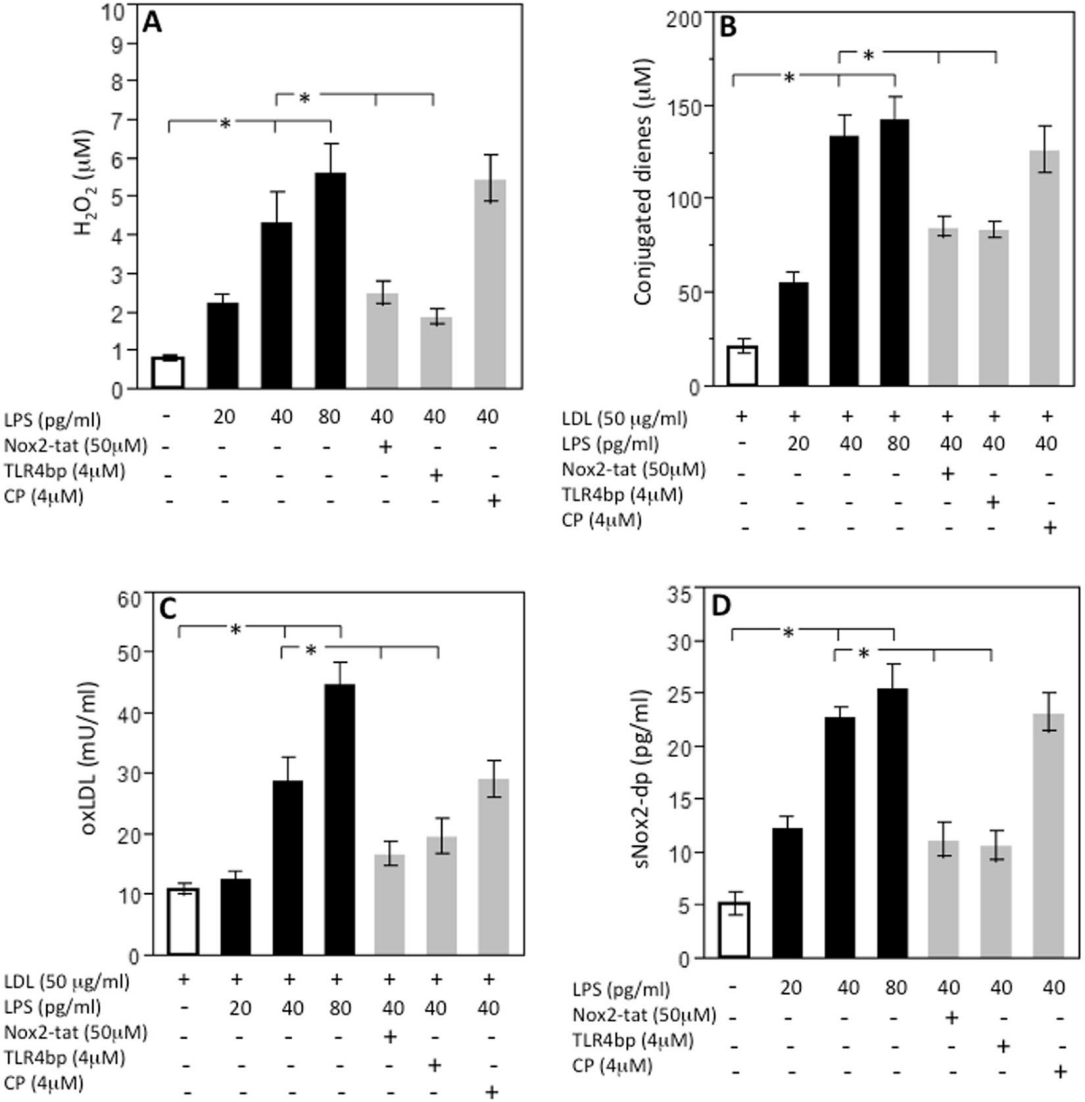
LPS-mediated oxidative stress in monocytes from healthy subjects. Lympho-monocytes from healthy subjects (HS; n = 5) were incubated with or without LDL (50 μg/ml) and stimulated with scalar concentrations of LPS (20–80 pg/ml) in presence or less of TLR4bp inhibitor (4 μM), Nox2-tat (50 μM) or control peptide (CP) (4 μM) for the evaluation of H_2_O_2_ production (**A**), conjugated dienes (**B**), oxLDL formation (**C**) and sNox2-dp (B) (*p < 0.01, Student’s t-test).

**Figure 4:**
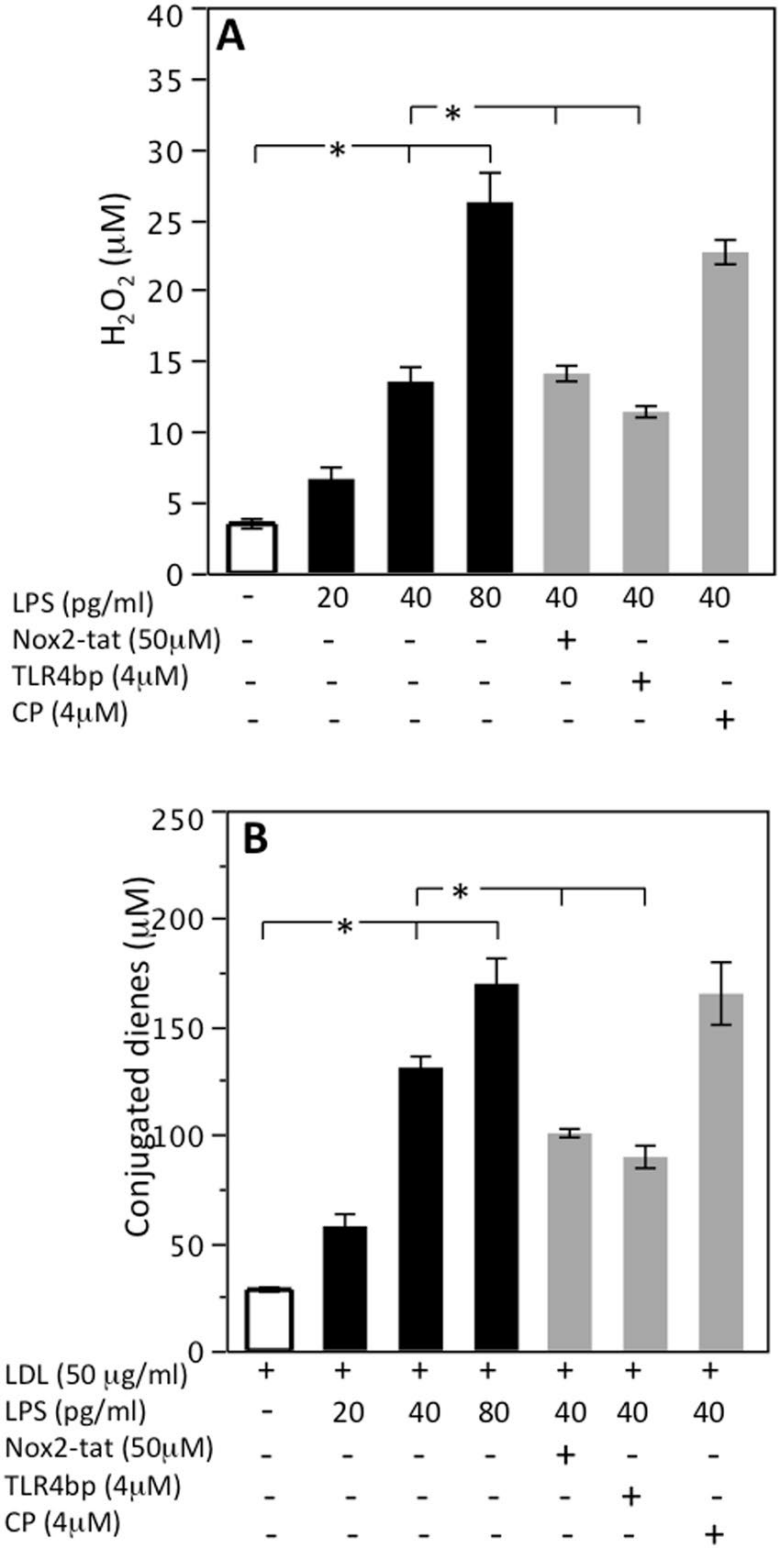
LPS-mediated oxidative stress in monocytes from patients with carotid stenosis. Lympho-monocytes from patients (HS; n = 4) were incubated with or without LDL (50 μg/ml) and stimulated with scalar concentrations of LPS (20–80 pg/ml) in presence or less of TLR4bp inhibitor (4 μM), Nox2-tat (50 μM) or control peptide (CP) (4 μM) for the evaluation of H_2_O_2_ (**A**) and conjugated dienes (**B**) (*p < 0.01, Student’s t-test).

As our data lead to hypothesize that LPS, once recognized by inflammatory cells, may behave as a pro-inflammatory molecule, we performed *in vitro* experiments to explore if LPS, at concentration detected in the plaque, was biologically active via interaction with TLR4. Concentration of LPS in carotid plaques was 42.3 ± 8.0 pg/ml and significantly correlated with the corresponding LPS circulating levels (r = 0.668, p < 0.05). Thus, we incubated monocytes from healthy subjects and patients with serial concentration of LPS (20–80 pg/ml) and found that LPS dose-dependently elicited a burst of oxidant species, as shown by a significant increase of hydrogen peroxide and conjugated dienes compared to control **(**Fig. 3, panels A and B and Fig. 4, panels A and B) and enhanced formation of oxidized LDL (Fig. 3C). All these effects were already statistically significant at 40 pg/ml LPS and blunted by cell incubation with a TLR4 inhibitor; conversely, no changes were detected with control peptide (Fig. 3A–C). To examine the mechanism related to LPS-mediated oxidative stress, we focused on the potential role of Nox2, which is among the most important cell producer of oxidant species^10^). Nox2 pertains prevalently to the innate immune system where its activity is crucial for bacterial killing^10^ but it also localizes in the artery wall where it is implicated in the athero-thrombotic process^11^. To assess the activity of Nox2, we measured, as previously described, a split-off product released in the medium as soluble Nox2-derived peptide (sNox2-dp) upon Nox2 activation^11^. The *in vitro* experiments demonstrated that downstream effect of cells incubated with LPS was characterized by a dose-dependent increase of sNox2-dp, an effect inhibited by cell incubation with TLR4 inhibitor (Fig. 3D). Further support to the role of Nox2 as trigger of LPS-mediated oxidative stress was provided by incubating cells with a Nox2 specific inhibitor, which, in fact, blunted the formation of hydrogen peroxide, conjugate dienes and oxidized LDL (Fig. 3A–D).

## Discussion

The study provides evidence that LPS from *Escherichia Coli* localizes in human atherosclerotic plaque but not in normal artery and may trigger inflammation via interaction with TLR4.

To investigate the relationship between LPS and atherosclerosis, we followed a previously described procedure consisting in examining atherosclerotic specimens from candidates to carotid endarterectomy (CEA)^12^. Carotid specimens displayed positivity for LPS in the area, which was characterized by intense positivity for antibody against macrophages. Co-localization of LPS immunostaining with that for an antibody against a specific macrophage epitope suggests specific LPS binding to macrophages detected in the atherosclerotic lesion; in accordance with this, we failed to detect LPS in the extracellular medium. At variance with macrophage-rich atherosclerotic lesion, normal arteries were negative for antibodies against either LPS or CD68, indicating that in absence of macrophages LPS is not implicated in atherosclerotic process unless macrophages have already infiltrated the artery wall.

Morphological changes are hallmarks of the macrophage inflammatory response. Formation of lamellipodia and filopodia, that are important for attachment to the extracellular matrix and ensuing cell adhesion and migration to inflammatory sites as well as cell size are markers of macrophage activation^13^. In this context, it is of particular interest the fact that positivity for LPS and TLR4 were detected essentially in atherosclerotic lesion characterized by activated macrophages, providing indirect evidence that LPS might act as stimulus for macrophage activation. This could occur via interaction with TLR4, which is a specific receptor for LPS on macrophage surface^14^ and was indeed detected by immunostaining in the same area where positivity for LPS and activated macrophages was evidenced. To corroborate this hypothesis, *in vitro* experiments were performed to establish if LPS, in the same range of concentration detected in atherosclerotic plaque, was able to activate human monocytes. We found that LPS dose-dependently activated monocyte via TLR4 activation with an intra-signaling mechanism involving up-regulation of Nox2, which is among the most important cellular producer of reactive oxidant species^15^. As this downstream effect resulted in formation of oxidized LDL, it could be arguable a role for LPS as molecule promoting oxidative stress at site of atherosclerotic lesion.

The study has implications and limitations. The mechanism accounting for LPS translocation from gut microbiota to atherosclerotic plaque was not fully addressed by the present study; however, changes in gut permeability might be a plausible mechanism as increased serum zonulin, which reflects enhanced gut permeability, significantly correlated with blood LPS. This hypothesis needs to be explored by directly analyzing gut permeability in patients with clinically overt atherosclerosis. We show that LPS from EC across the arterial wall and localizes in the atherosclerotic plaque but do not provide a direct evidence that LPS binds to TLR4 and activates macrophages *in situ*; however, our data provide a rationale to assess if lowering serum levels of EC-LPS from gut microbiota may be implicated in atherosclerosis progression. In this context Mediterranean diet, which is associated with reduced risk of atherosclerotic progression^16^, may be an interesting nutritional approach as adherence to this diet lowers circulating LPS concentration in patients prone to atherosclerotic complications^17^.

We have recently reported that in patients at risk of cardiovascular events circulating LPS concentration independently predicted myocardial infarction, stroke and cardiovascular death during a follow-up of about 3 years^17^. The significant correlation between concentration of LPS in blood and in atherosclerotic plaque provides a potential interpretation of this report and would suggest blood LPS concentration to be considered for clinical purpose as marker of atherosclerosis progression.

In conclusion, the positivity of LPS from EC in human carotid plaque provides further insight in the role of gut microbiota in atherosclerotic process and suggests LPS from EC as a novel trigger of the inflammation occurring in atherosclerosis.

## Methods

### Study design

We performed a cross-sectional study including 10 patients with critical stenosis of carotid artery (>70%) undergoing carotid endarterectomy (CEA). Fifteen control patients with a negative Doppler ultrasound for carotid plaques were also enrolled. Patients and controls were balanced for age (73.1 ± 8.9 vs. 70.7 ± 6.0, p = 0.433) sex (percentage of women was 20 vs. 26.7%, p = 0.702) and cardiovascular risk factors. Thus, all patients and controls were hypertensive and treated with antiplatelet drugs, 100% and 90% of patients and controls were treated with statins, respectively (p = 0.400). No significant difference in the distribution of diabetes was observed (30% of patients vs. 53.3% of controls, p = 0.414).

Cardiovascular risk factors, such as arterial hypertension^18^ and diabetes^19^ were defined according to international guidelines.

Patients with active cancer, systemic autoimmune of infectious diseases were excluded from the study. The study was conformed to the declaration of Helsinki and approved by the Ethical Committee of Sapienza University of Rome. All patients recruited gave written informed consent to participate in the study.

### Sample acquisition and tissue processing

Human carotid plaques were homogenized immediately after chirurgical excision, as previously described^20^, in 1 ml of phosphate buffer saline (PBS) containing 1% Triton X-100 and frozen overnight at −80 °C. The next day, non-dissolved fragments were removed by centrifugation at 12.000 rpm for 5 min and supernatant collected for biochemical analysis.

As negative control, the superior thyroid artery was obtained from same patients concomitantly to CEA; cross section of the superior thyroid artery was deemed necessary for an adequate exposure and dissection of carotid arteries.

A blood sample from antecubital vein was taken from patients and controls, who had fasted 12 hours. Blood was centrifuged at 300 × g for 10 minutes to obtain serum and stored at −80 °C.

### Measurement of Lipopolysaccharide

Lipopolysaccharide (LPS) levels in serum and in homogenates of carotid plaques were measured using a commercial ELISA kit (Product No. CSB-E09945h; Cusabio, Wuhan, China). Standards of LPS, purified from *Escherichia coli*, and samples were plated for 2 hours at room temperature onto a microplate pre-coated with the antibody specific for LPS. After incubation, samples were read at 450 nm. Values were expressed as pg/ml; intra-assay and inter-assay coefficients of variation (CVs) were 8% and 10%, respectively.

### Measurement of zonulin

Serum levels of zonulin were measured using a commercially ELISA kit (Product No. E-EL-H5560 Elabscience, Wuhan, China). Antibody specific for zonulin has been pre-coated onto a microplate and 100 µl of standards and patient sera samples were added and incubated 90 minutes at 37 °C. Then, a biotinylated detection antibody specific for zonulin and Avidin-Horseradish Peroxidase (HRP) conjugate was added to each microplate. The amount of zonulin was measured with a microplate auto-reader at 450 nm. Values were expressed as ng/ml. Both intra- and inter-assay CVs were <10%.

### Immunohistochemistry analysis

All samples were collected and fixed in 10% neutral buffered formalin, dehydrated in ethanol and embedded in paraffin. The sample was cut into 2 μm thick sections using a microtome. The slices were dewaxed in xylene, rehydrated through a graded series of ethanol solutions, washed in distilled water and PBS. Serial sections were subjected to antigen retrieval procedure by microwaving in 0.01 M sodium citrate buffer pH 6.0; endogenous peroxidase activity was blocked by incubation with 3% H2O2 in methanol for 15 min. The slices were incubated overnight at 4 °C with the following antibody: anti E. coli LPS antibody (1:100) (Product No. ab35654, Abcam, Cambridge, UK); anti-CD68 (Product No. NCL-CD68-KP1, Leica Biosystems, Wetzlar, Germany) was used to identify monocytes/macrophages; anti-CD31 antibody (Product No. PA0414, Leica Biosystems, Wetzlar, Germany) was used to identify angiogenesis; anti-CD34 antibody (Product No. PA0212, Leica Biosystems, Wetzlar, Germany) was used to identify endothelial vessel; anti-CD3 antibody (Product No. PA0122, Leica Biosystems, Wetzlar, Germany) was used to identify T-cells; anti-CD20 antibody (Product No. PA0359, Leica Biosystems, Wetzlar, Germany) was used to identify B-cells; TLR4 (1:100) (Product No. sc13593, Santa Cruz Biotechnology, Dallas, USA). After washing in PBS, the slices were processed as described in the protocol of kit Dako LSAB2 System-HRP (Product No. K0673, Santa Clara, USA). Slices were lightly counterstained with Haematoxylin, dehydrated in ethanol, cleared in xylene and mounted. Digital images were acquired using a digital System D-Sight (Menarini, Florence, Italy). The percentage of cells positive for LPS and TLR4 out of total macrophages (CD68 positive cells) were estimated by averaging positive cells in 3 regions using a 40X objective.

### *In vitro* study

#### Isolation of peripheral blood mononuclear cells (PBMCs)

Freshly taken EDTA-blood from healthy subjects and patients with carotid stenosis (n = 5, 3 males and 2 females age 44.4 ± 3.2 for healthy subjects and n = 5, 5 males age 75.6 ± 5.5 for patients) was diluted with PBS (1:4) and stratified carefully over 10 mL of Ficoll-Paque in 50 mL conical tube and then centrifuged at 1660 rpm for 30 minutes at 20 °C in a swinging-bucket rotor without brake. The mononuclear cell layer (lymphocytes and monocytes) at the interphase was aspired and transferred into a 50 mL conical tube. Finally, the tubes were filled with PBS and cell was washed 2 times by centrifugation at 1000 g for 10 minutes. Cells were incubated with Nox2ds-tat (50 µM, Product No. AS63818, Anaspec, California, USA) or TLR4bp (4 µM, Product No. NBP2–26244, Novus Biologicals, Colorado, USA) or control peptide (CP) (4 µM, Product No. NBP2–26244, Novus Biologicals, Colorado, USA) and added with or without serial LPS concentrations (20–80 pg/ml, Product No. L3015, Sigma Aldrich, Saint Louis, USA) in presence or less of 50 µg/ml of LDL. After 30 min incubation, samples were centrifuged for 3 minutes at 3000 rpm. Supernatants were stored at −80 °C for analysis of conjugated dienes, oxidized LDL (ox-LDL), H2O2 and sNox2dp.

#### Conjugated dienes

The standard oxidation assay was performed using a UV/VIS spectrometer (UV1800, AOE Instruments (Shanghai) Co., Ltd). Measurement of the 234 nm absorption was read in cell supernatant after stimulation and expressed as micromoles of conjugated dienes.

#### Hydrogen peroxide and oxidized LDL production

The hydrogen peroxide (H_2_O_2_) was evaluated by a Colorimetric Detection Kit (Product No. K034, Arbor Assays, Michigan, USA) and expressed as μM. Intra- and inter-assay CVs were 2.1% and 3.7% respectively. We measured oxLDL by ELISA commercial Kit (Product No. CSB-E07931h Cusabio, China). Values are expressed as mU/ml. Intra- and inter-assay CVs were <8% and <10%, respectively.

#### sNox2dp measurement

Extracellular levels of soluble Nox2-derived peptide (sNox2-dp), a marker of NADPH oxidase activation, were detected by ELISA as previously described^21^. The peptide was recognized by the specific monoclonal antibody against the amino acidic sequence (224–268) of the extra membrane portion of Nox2 (catalytic core of NADPH oxidase), which is released in the medium upon cell activation. Values were expressed as pg/ml; intra-assay and inter-assay CVs were 5.2% and 6%, respectively.

#### Statistical analysis

Categorical variables were reported as percentages, continuous variables as means ± standard deviation (SD). Comparisons between patients undergoing CEA and control patients were made by Student t-test (for continuous variables) or chi-square test (for categorical variables). Univariable correlation analysis was performed by the Pearson’s correlation coefficient. A value of p < 0.05 was considered as statistically significant. All analyses were performed with SPSS V.18.0 (SPSS Inc., Chicago, IL, USA).

Based on previous works^17,22^, the minimum sample size was computed with respect to a two-tailed Student t test for independent groups, considering 1) relevant difference of LPS levels to be detected between groups |δ| ≥ 30 with SDs homogeneous between the groups (SD = 20), and 3) type I error probability α = 0.05 and power 1-β = 0.90. This resulted in n = 10 for group.

## References

[1] Sanduzzi Zamparelli M (2016). The Metabolic Role of Gut Microbiota in the Development of Nonalcoholic Fatty Liver Disease and Cardiovascular Disease. Int. J. Mol. Sci..

[2] Tang WHW (2013). Intestinal Microbial Metabolism of Phosphatidylcholine and Cardiovascular Risk. N. Engl. J. Med..

[3] Saad MJA, Santos A (2016). & Prada, P. O. Linking Gut Microbiota and Inflammation to Obesity and Insulin Resistance. Physiology.

[4] Vors C (2015). Postprandial Endotoxemia Linked With Chylomicrons and Lipopolysaccharides Handling in Obese Versus Lean Men: A Lipid Dose-Effect Trial. J. Clin. Endocrinol. Metab..

[5] Lassenius MI (2011). Bacterial endotoxin activity in human serum is associated with dyslipidemia, insulin resistance, obesity, and chronic inflammation. Diabetes Care.

[6] Vink A (2002). *In vivo* evidence for a role of toll-like receptor 4 in the development of intimal lesions. Circulation.

[7] Lehr HA (2001). Immunopathogenesis of atherosclerosis: endotoxin accelerates atherosclerosis in rabbits on hypercholesterolemic diet. Circulation.

[8] Jaw JE (2016). Lung exposure to lipopolysaccharide causes atherosclerotic plaque destabilisation. Eur. Respir. J..

[9] Nocella, C. *et al*. Lipopolysaccharide as trigger of platelet aggregation via eicosanoid over-production. *Thromb*. *Haemost*. **117**, (2017). 10.1160/TH16-11-0857 28492699

[10] Kovács I, Horváth M, Lányi Á, Petheő GL, Geiszt M (2015). Reactive oxygen species-mediated bacterial killing by B lymphocytes. J. Leukoc. Biol..

[11] Pignatelli P (2011). Inherited Human gp91phox deficiency is associated with impaired isoprostane formation and platelet dysfunction. Arterioscler. Thromb. Vasc. Biol..

[12] Praticò D (1997). Localization of distinct F2-isoprostanes in human atherosclerotic lesions. J. Clin. Invest..

[13] Pi J (2014). Detection of lipopolysaccharide induced inflammatory responses in RAW264.7 macrophages using atomic force microscope. Micron.

[14] Bosisio D (2002). Stimulation of toll-like receptor 4 expression in human mononuclear phagocytes by interferon-gamma: a molecular basis for priming and synergism with bacterial lipopolysaccharide. Blood.

[15] Panday A, Sahoo MK, Osorio D, Batra S (2015). NADPH oxidases: an overview from structure to innate immunity-associated pathologies. Cell. Mol. Immunol..

[16] Estruch R (2013). Primary prevention of cardiovascular disease with a Mediterranean diet. N. Engl. J. Med..

[17] Pastori D (2017). Gut‐Derived Serum Lipopolysaccharide is Associated With Enhanced Risk of Major Adverse Cardiovascular Events in Atrial Fibrillation: Effect of Adherence to Mediterranean Diet. J. Am. Heart Assoc..

[18] ESH/ESC Task Force for the Management of Arterial Hypertension, G. *et al*. 2013 Practice guidelines for the management of arterial hypertension of the European Society of Hypertension (ESH) and the European Society of Cardiology (ESC): ESH/ESC Task Force for the Management of Arterial Hypertension. *J*. *Hypertens*. **31**, 1925–38 (2013). 10.1097/HJH.0b013e328364ca4c 24107724

[19] Authors/Task Force Members *et al*. ESC Guidelines on diabetes, pre-diabetes, and cardiovascular diseases developed in collaboration with the EASD: the Task Force on diabetes, pre-diabetes, and cardiovascular diseases of the European Society of Cardiology (ESC) and developed in collaboration with the European Association for the Study of Diabetes (EASD). *Eur*. *Heart J*. **34**, 3035–87 (2013). 10.1093/eurheartj/eht108 23996285

[20] Carnevale R (2007). LDL are oxidatively modified by platelets via GP91(phox) and accumulate in human monocytes. FASEB J. Off. Publ. Fed. Am. Soc. Exp. Biol..

[21] Pignatelli P (2010). Atorvastatin inhibits gp91phox circulating levels in patients with hypercholesterolemia. Arterioscler. Thromb. Vasc. Biol..

[22] Cangemi R (2016). Low-grade endotoxemia, gut permeability and platelet activation in community-acquired pneumonia. J. Infect..

